# Emerging therapeutic agents for genitourinary cancers

**DOI:** 10.1186/s13045-019-0780-z

**Published:** 2019-09-04

**Authors:** Kevin Zarrabi, Azzam Paroya, Shenhong Wu

**Affiliations:** 1 Department of Medicine, Stony Brook University Hospital, 9447 SUNY, Stony Brook, NY 11794-9447 USA; 2 Division of Hematology/Oncology, Department of Medicine, Northport VA Medical Center, Northport, NY USA

**Keywords:** Renal cell carcinoma, Prostate cancer, Bladder cancer, Immunotherapy

## Abstract

The treatment of genitourinary malignancies has dramatically evolved over recent years. Renal cell carcinoma, urothelial carcinoma of the bladder, and prostate adenocarcinoma are the most commonly encountered genitourinary malignancies and represent a heterogeneous population of cancers, in both histology and approach to treatment. However, all three cancers have undergone paradigm shifts in their respective therapeutic landscapes due to a greater understanding of their underlying molecular mechanisms and oncogenic drivers. The advance that has gained the most recent traction has been the advent of immunotherapies, particularly immune checkpoint inhibitors. Immunotherapy has increased overall survival and even provided durable responses in the metastatic setting in some patients. The early success of immune checkpoint inhibitors has led to further drug development with the emergence of novel agents which modulate the immune system within the tumor microenvironment. Notwithstanding immunotherapy, investigators are also developing novel agents tailored to a variety of targets including small-molecule tyrosine kinase inhibitors, mTOR inhibitors, and novel fusion proteins to name a few. Erdafitinib has become the first targeted therapy approved for metastatic bladder cancer. Moreover, the combination therapy of immune checkpoint inhibitors with targeted agents such as pembrolizumab or avelumab with axitinib has demonstrated both safety and efficacy and just received FDA approval for their use. We are in an era of rapid progression in drug development with multiple exciting trials and ongoing pre-clinical studies. We highlight many of the promising new emerging therapies that will likely continue to improve outcomes in patients with genitourinary malignancies.

## Introduction

Genitourinary (GU) malignancies encompass a heterogenous group of cancers pertaining to a specific anatomical and physiological function. There is incredible biological diversity among primary genitourinary malignancies [1]. Renal cell carcinoma (RCC); urothelial carcinoma of the bladder, ureter, and renal pelvis (UC); and prostate adenocarcinoma (PC) are the most commonly encountered histological subtypes within this group. Considering an annual morbidity of 225,000 patients and a mortality of over 56,000 patients per year in the USA from metastatic genitourinary malignancies, there remains an urgent and unmet need for new therapeutics [2].

We are witnessing a rapid evolution in diagnostic modalities with the emergence of novel biomarkers and clinical validation of new diagnostic tools. Further, there has been a paradigm shift in treatment guidelines with the rapid approval of a number of new agents for each respective tumor type. We have improved overall survival (OS) and progression-free survival rates (PFS), and the unprecedented success of the new armamentarium of immunotherapies and targeted therapies has been heralded a “revolution” in the treatment of GU malignancies. We anticipate the new survival data will be reflected in NCI SEER outcomes upon release of updated statistics.

The ongoing battery of clinical trials has benefited patients with approval of more treatment options, but has also created a degree of complexity to treatment regimens that clinicians must manage. Treatment plans for patients have become more variable as data has emerged supporting each respective agent, but fewer studies assessing the optimal sequence or combination of agents [3, 4]. Many of the open clinical trials entail investigation of both known agents repurposed for GU cancers that have shown success in other cancer models as well as novel compounds. Herein, we discuss key emerging therapeutic agents and therapeutic strategies involved in common GU tumors, specifically UC, RCC, and PC. We provide the biological rationale for the employment of the emerging agents as well as highlight some of the promising ongoing clinical trials.

## Bladder cancer

UC is the ninth most frequently diagnosed cancer worldwide, ranks 13th in death ranks, and is the most common cancer of the GU system [5, 6]. The median age of diagnosis is 73 years making bladder cancer a disease of the elderly [7]. The frailty and morbidity naturally inflicting the geriatric population pose a barrier to effective disease management as many patients are not candidates for current standard treatment [8]. Suboptimal eastern cooperative oncology group performance status in this age group is attributed to high incidences of renal insufficiency, neuropathy, hearing loss, and heart disease [9]. The diagnosis of bladder UC may be delineated between localized muscle-invasive, muscle-invasive bladder cancer (MIBC), and metastatic disease. MIBC poses a significant risk for metastasis [10]. Current standard of care treatment for MIBC entails neoadjuvant platinum-based chemotherapy followed by radical cystectomy [11]. OS rates with the standard approach remain less than ideal and complication rates are high [12, 13]. The options for locally advanced inoperable or metastatic UC remained limited as well, and these disease states carry a grim prognosis [10]. Historically, even with response to platinum-based chemotherapy, these patients carried a median OS of approximately 12–16 months [10, 14]. Further, approximately 50% of patients with MIBC are ineligible for treatment with platinum-based chemotherapy [15]. Until 2016, there were no approved post-platinum treatment agents available and second-line treatment options upon disease progression yielded a poor response rate of 10% [16, 17]. Since 2017, we have witnessed a number of landmark trials that have led to the approval of novel agents.

These include immune checkpoint inhibitors (CPI) as first-line treatment for patients with metastases who are not candidates for platinum-based therapy or who have progression of disease after platinum therapy [18–20]. The CPIs available for post-platinum salvage therapy are nivolumab, pembrolizumab, avelumab, atezolizumab, and durvalumab. Recently, the U.S. Food and Drug Administration (FDA) has granted accelerated approval to the tyrosine kinase inhibitor erdafitinib (Balversa) for patients with locally advanced or metastatic UC that have FGFR_2_ or FGFR_3_ genetic alterations and that have progressed on prior platinum-containing chemotherapy. We are now witnessing approval of a myriad of new treatment options with promising ongoing trials which will likely improve survival rates (Table 1).

**Table 1:** Emerging targets with clinical significance in urothelial carcinoma

Molecular target	Class	Trial	Disease setting	Agent	Experimental treatment	Study phase	Estimated completion
PD-1/PD-L1	CPI	NCT02807636 (IMvigor130)	Untreated mUC	Atezolizumab	Atezolizumab + (Carboplatin) (Gemcitabine) (Cisplatin)	Phase III	November 2020
		NCT02853305 (Keynote-361)	Untreated mUC	Pembrolizumab	Pembrolizumab + (Cisplatin) (Carboplatin) (Gemcitabine)	Phase III	May 2020
CTLA-4		NCT02516241 (DANUBE)	Untreated mUC	Tremelimumab	Durvalumab + tremelimumab or durvalumab monotherapy	Phase III	September 2019
CD122	Cytokine Modulation	NCT02983045 (PIVOT-2)	Advanced tumors (mUC)	NKTR-214	NKTR-214 + nivolumab or NKTR-214 + nivolumab + ipilimumab	Phase I/II	June 2021
		NCT03785925 (Pivot-10)	Cisplatin-ineligible, mUC patients with low PD-L1 expression	NKTR-214	NKTR-214 + nivolumab	Phase III	May 2022
		NCT03138889 (PROPEL)	Advanced tumors (mUC)	NKTR-214	NKTR-214 + pembrolizumab or NKTR-214 + atezolizumab	Phase I	May 2020
IL-7		NCT03513952	Advanced, inoperable or mUC	CYT107	CYT107 + atezolizumab	Phase I	December 2020
TGFβ + PD-1		NCT02517398	Advanced tumors (mUC)	MSB0011359C (M7824)	MSB0011359C (M7824)	Phase I	August 2020
CD137/4-1BB		NCT02845323	Cisplatin-ineligible MIBC	Urelumab	Urelumab + nivolumab or nivolumab monotherapy	Phase II	January 2020
OX40		NCT02554812 (JAVELIN Medley)	Advanced tumors (mUC)	PF-04518600	Avelumab Utomilumab PF-04518600 PD 0360324	Phase II	December 2020
		NCT03217747	Advanced tumors (UC)	PF-04518600	Avelumab UtomilumabPF-04518600 Radiation Cisplatin	Phase I/II	August 2023
IDO	T Cell Metabolism	NCT03361865 (Keynote-672) (ECHO307)	Cisplatin-ineligible UC	Epacadostat	Epacadostat + pembrolizumab	Phase III	September 2020
		NCT03374488 (Keynote-698)	Cisplatin-ineligible mUC	Epacadostat	Epacadostat + pembrolizumab	Phase III	August 2020
VEGF	TKI	NCT03133390	Cisplatin-ineligible mUC	Bevacizumab	Bevacizumab + atezolizumab	Phase II	January 2020
		NCT03272217	Cisplatin-ineligible untreated mUC	Bevacizumab	Bevacizumab + atezolizumab	Phase II	April 2021
cMET/VEGF		NCT02717156	Untreated mUC	EphB4-HSA	EphB4-HAS + pembrolizumab	Phase II	November 2020
		NCT03534804 (PemCab)	Cisplatin-ineligible mUC	Cabozantinib	Cabozantinib + pembrolizumab	Phase II	September 2023
		NCT03170960	Advanced tumors (UC)	Cabozantinib	Cabozantinib + atezolizumab	Phase I/II	December 2020
FGFR		NCT02546661 (BISCAY)	Previously-treated MIBC	AZD4547 MEDI4736 Olaparib AZD1775 Vistusertib AZD9150 Selumetinib		Phase Ib	March 2020
		NCT03123055 (FIERCE-22)	mUC	Vofatamab	Vofatamab + pembrolizumab	Phase I/II	September 2022
		NCT03473756 (FORT-2)	FGFR-positive mUC	Rogaratinib	Rogaratinib + atezolizumab	Phase Ib/II	July 2022
Nectin-4	ADC	NCT03288545 (EV-103)	mUC	Enfortumab Vedotin	Enfortumab vedotin + pembrolizumab	Phase I	September 2024
		NCT03219333 (EV-201)	mUC previously-treated with CPI	Enfortumab Vedotin	Enfortumab vedotin	Phase II	May 2025
		NCT02091999	Nectin-4-positive mUC	Enfortumab Vedotin	Enfortumab vedotin	Phase I	December 2020
		NCT03474107 (EV-301)	Previously-treated mUC	Enfortumab Vedotin	Enfortumab vedotin	Phase III	September 2021
HER-2		NCT03523572	mUC	Trastuzumab Deruxtecan (DS-8201a)	Trastuzumab Deruxtecan (DS-8201a)	Phase I/II	September 2020

All trial information obtained through publicly accessible clinicaltrials.gov

### Emerging immunotherapy through checkpoint inhibition

#### Checkpoint inhibition monotherapy

UC has long been considered an immunogenic tumor [21]. In fact, its immunogenicity has been harnessed as a treatment modality and UC has one of the longest track records of responsiveness to immunotherapy. Bacillus Calmette–Guérin was introduced as a treatment over 40 years ago [22]. Now, immune checkpoint blockade represents the most exciting sphere of emerging therapies for metastatic UC. The objective response rates (ORRs) for the approved post-platinum salvage therapy CPIs (nivolumab, pembrolizumab, avelumab, atezolizumab, and durvalumab) range from 15 to 31% [23]. At the moment, pembrolizumab is the only agent with an OS benefit shown by a randomized phase III study [20, 22].

With regards to CPI as first-line monotherapy in metastatic UC patients, both atezolizumab and pembrolizumab have been examined in patients. Atezolizumab, in the phase II IMvigor210 trial, and pembrolizumab, in the phase II KEYNOTE-052 trial, have both demonstrated clinically meaningful efficacy and objective responses [18, 24]. Both agents are now being studied independently in the phase III setting as monotherapies as well as with combination chemotherapy in previously untreated patients with locally advanced unresectable or metastatic disease. The trials are similarly designed, and the primary endpoints are PFS and OS. Atezolizumab in the IMvigor130 (NCT02807636) and pembrolizumab in the KEYNOTE-361 (NCT02853305) trials are currently underway with highly anticipated results. However, the progress of these trials may be muddled. Preliminary results have shown that the effect of these agents may be less effective than chemotherapy in certain patients, and monotherapy should be limited to patients with high PD-L1 expression. In fact, patients with low PD-L1 levels in the CPI arm of the trials had decreased survival compared to patients who received cisplatin- or carboplatin-based chemotherapy. Patients with low PD-L1 levels are no longer being enrolled in the KEYNOTE-361 or IMvigor130 trials [25, 26].

#### Checkpoint inhibition combination therapy

Novel CPI agents being investigated in the UC model include the IgG2 anti-CTLA-4 monoclonal antibody tremelimumab. Early phase I results in previously treated UC patients receiving combination tremelimumab/durvalumab demonstrated an ORR of 21% with a tolerable adverse event (AE) profile [27]. This combination therapy is now being investigated in the open-label phase III DANUBE trial (NCT02516241). The trial results are highly anticipated and are expected at the end of 2019.

### Emerging immunotherapy through cytokine modulation

#### Pegylated recombinant interleukin-2 therapy

Aside from CPI, alternative immunomodulation strategies in UC have been explored. There are two cytokine-based agonists under investigation at this time in metastatic UC. NKTR-214 is a novel agent being explored in the phase I/II setting. NKTR-214 is an investigational, first-in-class, CD122 preferential agonist that functions as a pegylated recombinant interleukin-2 (IL-2) with cellular effects in activation of CD8^+^ T and natural killer (NK) cells without unwanted expansion of T regulatory (Treg) cells in the tumor microenvironment [28]. The PIVOT-02 study is a multi-cohort phase I trial of NKTR-214 in combination with either nivolumab or ipilimumab/nivolumab therapy. PIVOT-02 includes patients undergoing first-line immunotherapy naïve and platinum-refractory metastatic UC patients (NCT02983045). Preliminary results presented at the 2019 American Society of Clinical Oncology (ASCO) Genitourinary Cancers Symposium were noteworthy for objective responses. The ORR was 48% in efficacy-evaluable patients, with 19% demonstrating a complete response (CR). ORR by immune-related RECIST was 52%. Treatment was well tolerated with only 15% of patients experiencing grade 3 treatment-related adverse events (TRAEs), and no patients experiencing grade 4/5 TRAEs. Of note, the PIVOT-02 trial demonstrates a thought-provoking phenomenon with regards to PD-L1 expression and may meet the urgent unmet need of novel treatments for patients whose tumors lack PD-L1 expression. The impressive ORR and CR were observed regardless of baseline PD-L1 expression. Moreover, 70% of patients who were PD-L1–negative prior to treatment converted to PD-L1–positive expressers after exposure to combination therapy. All PD-L1–positive patients maintained their PD-L1 positivity [29]. These data represent a remarkable breakthrough in immunotherapy treatment as lack of PD-L1 expression remains a barrier to optimal treatment for many patients. It serves as a proof-of-principle in novel approaches to induction of PD-L1 expression. The exact mechanism of PD-L1 modulation remains unclear, but among patients who underwent on-treatment biopsy versus a baseline biopsy, there was influx of CD8^+^ T cells. NKTR-214 may trigger a more vigorous local immune response within the tumor microenvironment [30]. These preliminary trial results have prompted the more expansive phase II PIVOT-10 study evaluating NKTR-214 in combination with nivolumab in locally advanced or metastatic UC cisplatin-ineligible patients with low PD-L1 expression (NCT03785925). An alternative yet similar trial in premise to PIVOT-02 is the phase I PROPEL trial (NCT03138889). PROPEL is investigating atezolizumab in combination with dose escalations of NKTR-214 in patients with platinum-resistant mUC. Another cytokine agonist being explored in the phase II setting is CYT107, a glycosylated recombinant IL-7 agent. Platinum-resistant and cisplatin-ineligible mUC patients are being subject to treatment with intramuscular CYT107 with atezolizumab versus atezolizumab monotherapy (NCT03513952).

#### TGFβ inhibition therapy

Knudson and colleagues have designed a bidirectional fusion protein integrating both CPI and immune cytokine TGFβ inhibition. Such a novel compound serves as the first in a new class of agents that regulate immune suppression in the tumor microenvironment in distinct yet complementary ways [31]. The agent, named M7824, comprises of the extracellular domain of TGFβRII and is linked with the C-terminus of the human anti-PD-L1 heavy chain. Pre-clinical work has been promising, and phase I enrollment is currently open for locally advanced solid tumors (NCT02517398) [32].

#### 4-1BB inhibition therapy

A cytokine modulator with early phase efficacy in UC is utomilumab, an investigational fully humanized IgG2 monoclonal antibody which acts as a 4-1BB agonist. 4-1BB is a receptor ubiquitous to T cells (CD4^+^, CD8^+^, NK, and memory T cells) and signals for T cell expansion. In pre-clinical models, utomilumab has demonstrated anti-tumor activity via T cell–mediated immune responses [33]. The phase Ib KEYNOTE-036 trial employing utomilumab with pembrolizumab in advanced tumors including UC revealed no dose-limiting toxicities and an ORR of 26.1% [34]. Further studies investigating utomilumab in UC patients are expected. Another 4-1BB immunotherapeutic under investigation is urelumab, a fully human monoclonal IgG4k antibody agonist of CD137/4-1BB [35]. Downstream effects include tumor necrosis factor (TNF) signaling cascade activation with effect on activated T and NK cells. Urelumab is under investigation in a phase II study in combination with nivolumab as neoadjuvant therapy in cisplatin-ineligible patients (NCT02845323).

#### OX40 inhibition therapy

Yet another immunomodulating target with a potential role in UC is OX40. OX40 are TNF proteins that are expressed on activated CD4^+^ and CD8^+^ T cells. OX40 signaling promotes T cell proliferation and survival, enhances cytokine production, and modulates cytokine receptor signaling, effectively augmenting the innate and adaptive components of immunity [36]. Additionally, OX40 activation downregulates Treg activity, further amplifying the process [37]. It is no surprise that investigators have developed targeted antibodies to OX40 for cancer therapy. PF-04518600, MOXR0916, and GSK3174998 are all novel agents that are being explored in advanced cancers in combination therapies. NCT02315066 is an early stage dose-escalation trial testing utomilumab with PF-04518600 in patients with advanced cancer, including UC patients. Results revealed no drug-related deaths, dose-limiting toxicities, or suspected unexpected serious TRAEs [38]. Preliminary results show an ORR of only 5.4%, but the stable disease rate was 29.7%. The stable disease rate in the UC cohort was 50% [39]. Although a small sample size, the staggering results have led to the phase I/II trials, JAVELIN Medley and NCT03217747, employing PF-04518600 with either CPIs, immunomodulators, cisplatin, or radiotherapy. Both studies are actively recruiting patients.

### Emerging immunotherapy through IDO inhibition

Another immunomodulating molecular target is indoleamine-2,3-dioxygenase (IDO). IDO is an intracellular enzyme with downstream effects on T cell activity. Specifically, IDO activation induces tryptophan degradation and kynurenine production which in turn downregulates T effector cells and upregulates T regulatory cell activity [40]. In effect, IDO activity enhances the immunosuppressive effects of the tumor in its microenvironment and provides the substrate for unregulated tumor growth [41]. IDO has served as a target for cancer therapies and is under investigation in patients with metastatic UC treated after platinum chemotherapy. An oral IDO inhibitor, epacadostat, has been investigated in combination with pembrolizumab in the phase I/II ECHO-202/KEYNOTE-037 trial, and the results are encouraging. The subgroup analysis of the metastatic UC patients revealed an ORR of 35% and a CR of 8% in the treatment arm. PD-L1–positive patients experienced an ORR of 64%, while PD-L1–negative patients experienced a 13% ORR. The addition of epacadostat did not lead to any greater incidence of grade 3/4 TRAEs compared to the pembrolizumab monotherapy group [42, 43]. The successful results of ECHO-202/KEYNOTE-037 springboarded this combination therapy into two phase III trials; KEYNOTE-672 comparing epacadostat or placebo with pembrolizumab in untreated, cisplatin-ineligible patients with advanced UC (NCT03361865), and KEYNOTE-698, which has the same experimental and treatment arms in patients with advanced UC who have failed first-line platinum chemotherapy (NCT03374488) [44]. Both trials are currently ongoing with no preliminary data available. There is currently one additional IDO inhibitor being studied in advanced UC patients. NCT03192943 is an industry-sponsored phase I trial investigating the safety and tolerability profile of agent BMS-986205 given in combination with nivolumab in patients with advanced tumors. Larger trials employing BMS-986205 are expected in the near future.

#### Emerging targeted therapy

The advent of immunotherapy has been a breakthrough in the treatment landscape of UC; however, durable responses are only observed in a minority of patients, and response rates are approximately 20% in the first- and second-line settings and beyond [45], fewer than that benefit from long-term remissions [44]. Similarly, targeted therapies have historically also provided poor response rates and targeted therapy monotherapy has had little success in the metastatic UC model. However, preclinical data suggest that several known anti-angiogenesis agents and tyrosine kinase inhibitors (TKIs) may augment the effects of immunotherapeutics in the tumor microenvironment [46]. As such, there are numerous ongoing trials investigating known and novel agents in conjunction with immunotherapy in UC.

#### Vascular endothelial growth factor receptor inhibition therapy

Vascular endothelial growth factor (VEGF) remains an optimal target as studies have shown that elevated urinary and serum VEGF levels in patients with UC suffer poorer prognoses and harbor more aggressive tumors [47, 48]. A VEGF-A inhibitor, bevacizumab, has already demonstrated pre-clinical efficacy in combination with PD-L1 inhibition in RCC [49]. There are two ongoing phase II trials investigating bevacizumab with atezolizumab in cisplatin-ineligible patients and previously untreated mUC, respectively (NCT03133390, NCT03272217). A second agent targeting VEGF is ramucirumab, a monoclonal antibody (mAB) targeting VEGFR_2_. Ramucirumab was recently tested in combination with pembrolizumab in a phase I multi-cohort study in UC patients with prior progression on platinum-based systemic therapy. Combination therapy treated patients suffered tolerable TRAEs and demonstrated objective anti-tumor activity [50]. In the phase III setting, ramucirumab has been paired with traditional chemotherapy in 530 patients with post-platinum mUC in the RANGE trial. Remarkably, the patients in the experimental arm with ramucirumab and docetaxel benefited from a mPFS of 4.07 months [95% CI 2.96–4.47] versus 2.76 months [95% CI 2.60–2.96] (hazard ratio [HR] 0.757, 95% CI 0.607–0.943; *p* = 0.0118). An objective response was achieved in 24.5% (95% CI 18.8–30.3) of patients allocated to ramucirumab and 14.0% (95% CI 9.4–18.6) assigned to placebo. These results are most noteworthy as they represent the first treatment regimen to demonstrate a PFS advantage over chemotherapy in the post-platinum setting. Moreover, these data further validate VEGF_2_ inhibition as a therapeutic avenue in metastatic UC [51]. The RANGE trial will likely set a precedent for future trial development. Other strategies towards VEGF inhibition include a novel recombinant EphB4-HSA fusion protein. EphB4-HSA is being investigated in combination with pembrolizumab in previously untreated stage IV UC as a phase II study (NCT02717156). The study is in the recruitment stage. Lastly, cabozantinib is being investigated in a number of trials. Cabozantinib is a small-molecule TKI with target receptors to RET, KIT, AXL, FLT3, MET, and VEGFR_2_ that was recently approved for metastatic RCC (mRCC) in the second-line setting after the METEOR trial [52]. Cabozantinib has demonstrated early success in combination with CPI. The UC cohort in a phase I trial of patients with GU malignancies naïve to CPI demonstrated a mPFS of 12.8 months (95% CI 1.8–N/A) and an OS rate of 70.2% (95% CI 44.4–85.8%) [53, 54]. Cabozantinib is also being investigated with other CPIs, including pembrolizumab and atezolizumab, respectively (NCT03534804, NCT03170960).

#### Nectin inhibition therapy

Nectins represent an interesting and novel therapeutic target for UC. Nectin-4 is a transmembrane polypeptide involved in cell-adhesion and has a role in tumor proliferation and angiogenesis [55]. Translational researchers have used suppression subtractive hybridization on UC pathological specimens and shown high mRNA expression of nectin-4 in bladder cancer [56]. Drug discovery efforts have produced enfortumab vedotin, a novel antibody-drug conjugate (ADC) composed of a mAB to nectin-4 bound to a potent cytotoxic microtubule inhibitor, monomethyl auristatin E. ADCs are a unique class of agents which combine highly specific mABs with toxic drugs [57]. In a phase I dose-escalation study investigating enfortumab vedotin in 68 patients with metastatic UC, the ORR was 41%, and the disease control rate was 72%. These staggering results also included a highly tolerable toxicity profile with only 9% of patients suffering grade 3/4 TRAEs [58, 59]. These data garnered much excitement, and enfortumab vedotin was granted breakthrough therapy status by the FDA for patients with metastatic UC previously treated with a checkpoint inhibitor. Three subsequent phase I–III trials employing enfortumab vedotin were designed soon thereafter, the EV-103 [60], EV-201(NCT03219333), and EV-301 trials [61]. At the 2019 ASCO annual meeting, the results from the single-arm phase II EV-201 trial was reported. Enfortumab vedotin induced a 44% response rate in patients with locally advanced or metastatic UC. Twelve percent of those patients are currently experiencing a complete response. These results are staggeringly similar to the phase I trial results, which strengthens the enthusiasm for the agent. The EV-201 enrolled patients who had been treated with platinum-based chemotherapy and/or checkpoint inhibitors. The mOS was 11.7 months (95% CI 9.1–N/A), mPFS was 5.8 months (95% CI 4.9–7.5), median duration of response (mDOR) was 7.6 months (range, 0.95–11.30+), all with a well-tolerated adverse effect profile [62]. Enfortumab vedotin is now the first novel therapeutic agent to demonstrate clinical benefit in patients who progressed after CPI therapy. The phase III EV-301 and-201 trials are currently underway.

#### Human epidermal growth factor receptor inhibition therapy

The human epidermal growth factor receptor (HER) family has been widely investigated, and its targeting is embedded as a cornerstone in the treatment of breast and gastrointestinal malignancies [63]. HER2 (Erb2) activation results in tumor cell growth, proliferation, and even chemotherapy resistance [64]. HER2 expression in UC has been well established and UC has one of the highest rates of HER2 expression of any solid tumor [65]. However, the UC patient population has not benefited from HER2 targeting as the data remains unclear to any clinical efficacy in unselected UC patients. A novel ADC has been developed, trastuzumab deruxtecan, and is being investigated in combination with nivolumab in a multicohort phase I trial inclusive of patients with UC (NCT03523572).

#### Fibroblast growth factor receptor inhibition therapy

The fibroblast growth factor (FGF) pathway is yet another well-elucidated tyrosine kinase (TK) signaling pathway implicated in tumorigenesis and has a high mutational expression rate in UC [16]. As the first TKI approved in UC therapy, the ORR for erdafitinib was 32.3% with 2.3% having a CR in a clinical trial that included 87 patients with advanced bladder cancer with FGFR_2_ or FGFR_3_ genetic alterations [66]. Major side effects include vision changes associated with retinal disorder and hyperphosphatemia. BGJ-398 is a pan-FGFR inhibitor that has been studied in patients with metastatic UC. In a phase Ib trial inclusive of previously treated metastatic UC patients with FGFR_3_ alterations, BGJ-398 demonstrated a disease control rate of 64.2% [67]. Such a dramatic anti-tumor response has prompted development of a multitude of trials investigating anti-FGF therapies in metastatic UC. Most notable is the BISCAY phase I study, an umbrella trial of durvalumab in combination with a potent and selective novel FGFR inhibitor, AZD4547. The trial is inclusive only of patients presenting with FGFR_3_ mutations (NCT02546661). Vofatamab (B-701) is yet another novel FGFR_3_ inhibitor, and it is being studied in combination with pembrolizumab in the FIERCE-22 international phase I/II trial in metastatic UC (NCT03123055). Rogaratinib (BAY1163877), a novel pan-FGFR inhibitor by Bayer, is being trialed in various solid tumors, including metastatic UC in the FORT-2 trial as combination therapy with atezolizumab (NCT03473756) [68].

## Renal cell carcinoma

RCC is a heterogeneous disease with the majority of cases categorized into one of two major histological subtypes; 80% are clear cell RCC (ccRCC) and 20% are non-clear cell RCC (nccRCC) [69]. RCC is a commonly encountered GU malignancy with over 320,000 patients diagnosed annually and an annual death toll of over 140,000 people worldwide. More concerning, the annual incidence has risen over the past 10 years and now accounts for nearly 4% of new cancer diagnoses in the USA [70, 71]. Clinicians have been challenged with the peculiarity of RCC, as it harbors features that contrast those of prototypical cancers. RCC often lacks features of classic carcinomas and its mechanisms of metastasis have been difficult to combat [72]. One-quarter of patients diagnosed with organ confined disease suffer from recurrence with metastases in their disease course. Prior to 2005, there was very little progress in treatment advances for mRCC and the mainstay of therapy remained high-dose interleukin-2 (HDIL-2) and interferon-alpha (IFN-α) after FDA approval in the 1990s [73]. The tumor had proven resistant to radiotherapy, hormonal therapy, and conventional chemotherapies [74, 75]. The cytokine-based therapy was non-specific and was associated with significant systemic toxicity, and responses were modest [76]. To this end, mRCC has been a difficult cancer to treat and has purported a poor prognosis [75, 77].

However, we have since strengthened our understanding of the molecular mechanisms behind RCC tumorigenesis. The landmark discovery of the *VHL* tumor-suppressor gene and the observation that *VHL* is mutated in up to 90% of patients with ccRCC has helped elucidate the molecular interplay in the RCC tumor microenvironment [78]. Targeting signaling molecules downstream to VHL has identified targets for therapeutics. These include VEGF_1-3_, mTOR, PDGFRα, MET, FGFR_1-4_, RET, KIT, and AXL. Moreover, the emergence of immunotherapy has further expanded the armamentarium of agents approved for RCC. The efficacy of HDIL-2 and IFN-α served as a proof-of-principle of the immunogenic potential of RCC for drug development, and RCC was one of the first tumor models to demonstrate objective tumor responses to CPI. In fact, PD-L1 expression directly correlates with tumor stage, Fuhrman grade, sarcomatoid differentiation, and inversely correlates with patient survival in mRCC patients [79]. It is no surprise that both targeted therapies and CPIs have dominated the therapeutic landscape in RCC.

### Current therapeutic landscape

The first phase I study evaluating nivolumab was conducted in select advanced tumors, which included RCC [80]. Since that study, the ensuing 5-year period witnessed rapid development and completion of phase I (CheckMate016) through phase III (CheckMate 025) randomized control trials leading to FDA approval of the agent for second-line treatment in those who fail VEGFR-targeting therapies [81]. The success of CPI in the second-line setting laid the foundation for experimental design investigating dual CPI (PD-1 and CTLA-4 blockade) in the first-line setting. The highly anticipated CheckMate-214 trial results were released in 2018. Nivolumab with ipilimumab combination significantly improved ORR and OS compared to sunitinib in patients with intermediate-and poor-risk disease. There was no significant difference in mPFS [82]. Soon thereafter in April 2018, nivolumab with ipilimumab gained FDA approval and now holds NCCN category one recommendation for intermediate- and poor-risk previously untreated mRCC patients [83].

A number of alternative CPIs are showing success in the phase III setting with recent FDA approval. In fact, in February 2019, in the same issue of the New England Journal of Medicine, results from two highly anticipated trials were released. The Javelin Renal 101 study was a phase III randomized control trial enrolling previously untreated mRCC patients and offered either avelumab plus axitinib or sunitinib monotherapy. The mPFS for the combination treatment was 13.8 months versus 8.4 months with sunitinib (HR 0.69; 95% CI 0.56 to 0.84; *p* < 0.001). The OS was 11.6 months versus 10.7 months. Toxicities between the two groups were comparable [84]. In a similarly designed open-label phase III trial, 861 previously untreated mRCC patients were randomly assigned to receive pembrolizumab plus axitinib or sunitinib monotherapy (KEYNOTE-426). mPFS was 15.1 months in the combination group and 11.1 months in the sunitinib group (HR for disease progression or death, 0.69; 95% CI 0.57 to 0.84; *p* < 0.001). The most impressive data set from the study was the OS data; 89.9% in the pembrolizumab–axitinib group and 78.3% in the sunitinib group were alive at 12-month follow-up (HR for death, 0.53; 95% CI 0.38 to 0.74; *p* < 0.0001). This data represents the lowest HR recorded among frontline therapy trials [85]. Of note, KEYNOTE-426 was remarkable in that the benefits were observed across all International Metastatic Renal Cell Carcinoma Database Consortium risk groups. Once again, toxicities were comparable between the two arms [86]. These two studies led to recent FDA approval of avelumab or pembrolizumab with axitinib in the first-line treatment of advanced RCC. The final high-impact phase III trial of 2019 with CPI in mRCC was the IMmotion-151 trial, the first randomized phase III study combining a PD-L1/PD-1 pathway inhibitor with an anti-VEGF agent in mRCC. Treatment-naïve mRCC patients were randomized to receive atezolizumab plus bevacizumab or sunitinib. mPFS favored the atezolizumab plus bevacizumab combination arm in the PD-L1–positive patients (11.2 months versus 7.7 months, HR 0.74; 95% CI 0.57 to 0.96, *p* = 0.02) as well in the intention to treat patients (HR 0.83; 95% CI 0.70 to 0.97, *p* = 0.02). OS data were not reached at the interim analysis and are pending. Atezolizumab plus bevacizumab-treated patients suffered fewer grade 3/4 TRAEs compared to that of sunitinib, 40% versus 54%, respectively (NCT02420821) [87]. The compelling data from these three major randomized control trials are expected to change first-line treatment practices for mRCC.

Current FDA-approved first-line agents are the TKI monotherapy with axitinib, cabozantinib, pazopanib, and sunitinib; combination CPI therapy with nivolumab and ipilimumab, pembrolizumab, and avelumab; combination TKI-CPI with axitinib and avelumab; mTOR inhibition with temsirolimus; and cytokine therapy with HDIL-2. The optimal sequencing of therapies has been heavily debated with few consensus guidelines in the literature. Updated NCCN guidelines recommend axitinib and pembrolizumab, pazopanib, or sunitinib as preferred first-line agents in favorable risk patients. The recommendation further includes ipilimumab and nivolumab, axitinib and pembrolizumab, or cabozantinib monotherapy for poor/intermediate risk patients [88]. Second-line therapy may employ monotherapy with nivolumab, axitinib, pazopanib, sunitinib, cabozantinib, sorafenib, HDIL-2, everolimus, temsirolimus, or bevacizumab or combination therapy with ipilimumab and nivolumab, lenvatinib and everolimus, axitinib and pembrolizumab, or axitinib and avelumab. The tremendous number of available treatment agents has led to variability among therapy regimens between patients. There are few studies to date reconciling the data from independent studies. In previous years, clinicians were challenged with a lack of therapies and overwhelming toxicities, whereas todays’ landscape offers an abundance of therapies with complex data supporting them [3]. The prolific rate of drug development continues, and emerging therapies target a vast array of molecular mechanisms and will further advance the treatment options available to patients with RCC (Table 2).

**Table 2:** Emerging targets clinical significance with ongoing clinical trials in RCC

Molecular target	Class	Trial	Disease setting	Agent	Experimental treatment	Study phase	Estimated completion
VEGF	TKI	NCT01253668	Previously-treated mRCC	Brivanib		Phase II	Completed*
TGFβ		NCT01806064	Previously-treated mRCC	Endoglin	Axitinib + endoglin	Phase I/II	June 2019
cMET		NCT02761057 (PAPMET)	Papillary mRCC	Cabozantinib Cabozantinib S-malate Crizotinib Savolitinib Sunitinib		Phase II	January 2021
		NCT03091192	MET-driven papillary mRCC	Savolitinib		Phase III	August 2019
CCR4	Cytokine modulator	NCT02281409	Advanced tumors (mRCC)	Mogamulizumab		Phase I/II	October 2019
		NCT02946671	Pre-operative advanced tumors (mRCC)	Mogamulizumab	Mogamulizumab + nivolumab	Phase III	March 2020
HIF2-α	Small-molecule inhibitor	NCT02293980	Previously-treated mRCC	PT2385	PT238 monotherapy or PT2385 + nivolumab or PT2385 + cabozantinib	Phase I	August 2020
		NCT03108066	VHL disease-associated ccRCC	PT2385		Phase II	September 2022
PD-L1	CPI	NCT02420821 (IMmotion-151)	Previously-untreated mRCC	Atezolizumab	Atezolizumab + bevacizumab	Phase III	December 2021
		NCT02811861 (Keynote-581/CLEAR)	Previously-untreated mRCC	Pembrolizumab	Pembrolizumab + lenvatinib	Phase III	February 2021
		NCT03141177 (CheckMate 9ER)	Previously-untreated mRCC	Nivolumab	Nivolumab + cabozantinib	Phase III	April 2023
		NCT03149822	mRCC	Pembrolizumab	Pembrolizumab + cabozantinib	Phase I/II	June 2020
CTLA-4		NCT02762006	Previously-untreated localized RCC	Neoadjuvant tremelimumab	Neoadjuvant tremelimumab + durvalumab	Phase I	April 2019**
		NCT02626130	Previously-treated mRCC	Tremelimumab	Tremelimumab or tremelimumab with cryoablation	Pilot study	March 2022
Autologous DCs	Tumor vaccine	NCT02432846 (MERECA)	mRCC	Intuvax	Intuvax + nephrectomy+ sunitinib	Phase II	August 2019

All trial information obtained through publicly accessible clinicaltrials.gov

*Announcement of study results pending

**Remains active

### Emerging novel anti-angiogenesis agents

#### Vascular endothelial growth factor receptor inhibition therapy

Due to the redundancy of anti-angiogenesis targets shared among solid tumors, investigators are able to repurpose agents already developed for other tumors to the RCC model. Brivanib is an investigational, anti-angiogenesis oral TKI previously developed for the treatment of hepatocellular carcinoma, although it currently holds no FDA approval for clinical use in any setting. Brivanib inhibits VEGFR_2,_ FGFR, and downregulates cyclin D1, Cdk-2, Cdk-4, cyclin B1, and phospho-c-Myc [89]. Brivanib is under investigation in mRCC in a single-arm, phase II study in patients with refractory metastatic disease (NCT01253668). The study is complete and the announcements of results are pending.

#### Activin receptor-like kinase 1 inhibition therapy

Activin receptor-like kinase 1 (ALK) is a TK member of the TGFβ superfamily. Interestingly, its role as a signaling molecule for angiogenesis is independent of VEGF and FGFR signaling [90]. Dual blockade of VEGF and ALK signaling pathways with combination therapies was a novel and intriguing approach to anti-angiogenesis. To this end, Voss and colleagues developed the DART trials, investigating ALK inhibitor dalantercept in combination with axitinib in patients with mRCC after TKI therapy. Phase I results were promising, combination dalantercept and axitinib was well tolerated, the ORR was 25%, and disease control was reported at 57% [91]. However, the recently reported results from the phase II DART trial in which 124 patients were randomized 1:1 to receive axitinib plus dalantercept versus axitinib plus placebo are less encouraging. There has no mPFS benefit in the dalantercept plus axitinib group and the ORR was 19.0% (95% CI 9.9–31.4%) in the dalantercept plus axitinib group and 24.6% (15 of 61 patients; 95% CI 14.5–37.3%) in the placebo plus axitinib group. Although well tolerated, the combination therapy was considered a failure [92]. At this time, Acceleron pharmaceuticals has discontinued development of dalantercept for mRCC [93]. More promising are ongoing trials investigating endoglin inhibition. Endoglin is a homodimeric TGFβ co-receptor that is upregulated in the setting of *VHL* mutation and HIF1-α overexpression. It is essential for angiogenesis. Investigators have identified the endoglin glycoprotein as a novel non-VEGF angiogenesis pathway that has the potential to complement VEGF-targeted therapy [94]. As such, Choueiri and colleagues have recently employed a chimeric IgG1 mAB targeting endoglin with axitinib in patients with mRCC. The results of a phase Ib trial were recently released, and the combination therapy demonstrated both clinical activity with partial responses in 29% of patients with no dose-limiting toxicity in a VEGF inhibitor-refractory population [95]. A multicenter, randomized phase II trial investigating combination therapy has recently completed accrual (NCT01806064).

#### CCR4, cMET, and HIF2-α inhibitor inhibition therapy

Novel emerging therapeutic agents include CCR4, cMET, and HIF2-α inhibitors. CCR4 has molecular implications in angiogenesis, and its inhibition has shown anti-cancer properties [96, 97]. Mogamulizumab is a mAB inhibitor of CCR4, and it is concurrently being investigated in both the phase I and II settings in advanced cancers including mRCC to assess safety and tolerability (NCT02281409) as well as clinical efficacy when in combination with nivolumab (NCT02946671). The identification of HIF accumulation as a sequela of *VHL* mutation in RCC provides the rationale for drug development towards HIF inhibition [98]. PT2385 is a novel small-molecule inhibitor of HIF2-α. Both phase I and II trials are actively recruiting patients with advanced ccRCC (NCT02293980, NCT03108066). Lastly, we have witnessed cMET inhibition transition to the forefront of mRCC drug development. The objective responses and OS benefit seen with cabozantinib therapy served as a proof-of-principle that cMET may have an in vivo role in mRCC [3]. Four-small molecule inhibitors of cMET are currently under investigation: crizotinib, volitinib, foretinib, and savolitinib. The EORTC 90101 CREATE trial has demonstrated safety and tolerability of crizotinib mRCC patients with MET amplification [99]. Trials including these agents are ongoing (NCT02761057, NCT03091192).

### Emerging mTOR-autophagy inhibition combination therapy

A well-established driver of tumorigenesis and angiogenesis is the mammalian target of rapamycin (mTOR), a serine/threonine kinase member of the PI3K family [100]. mTOR was one of the first focuses of targeted therapy research in RCC, and there are two FDA-approved agents which have roles in both the first-line and refractory settings. Temsirolimus and everolimus are both viable options in clinical practice; however, clinical benefits are often modest compared to VEGF inhibitors. In fact, first-line temsirolimus is only recommended in patients with a poor prognosis stratified by the MSKCC prognostic model, and everolimus has failed to demonstrate benefit over other agents in the first line-setting [101, 102]. As such, the body of literature pertaining to mRCC has primarily focused on alternative treatment approaches and mTOR inhibition is often considered a less efficacious treatment choice [52, 78]. There is ongoing research on ways to augment mTOR inhibition. One such approach is combination of autophagy inhibition with mTOR blockade. Autophagy is the intracellular mechanism by which cells digest metabolic substrate and recycle macromolecules and nutrients. Interestingly, due to the high metabolic demand of cancer cells, autophagy is inherent to cancer cell survival and proliferation [103]. Amaravadi and colleagues are leading efforts to integrate autophagy inhibitors into oncology practice. Chloroquine is known to inhibit autophagic flux by decreasing autophagosome-lysosome fusion [104]. In a phase I/II trial in patients with advanced RCC, everolimus was combined with maximum dose hydroxychloroquine for assessment of safety and tolerability as well as ORRs. Hydroxychloroquine at 600 mg twice daily with 10 mg daily everolimus was tolerable, and the primary endpoint of > 40% 6 month PFS was met [105]. Autophagy inhibition has been a successful strategy in vitro and in vivo, and there is optimism that synergistic cell death with mTOR and hydroxychloroquine will succeed in larger trials.

### Emerging immunotherapy through checkpoint inhibition

#### Checkpoint inhibition and anti-angiogenesis combination therapy

Two highly anticipated phase III trials in the pipeline are KEYNOTE-581/CLEAR and the CheckMate 9ER trial, neither of which have mature data available at this time. KEYNOTE-581/CLEAR is a multicenter, open-label, phase III study evaluating pembrolizumab plus lenvatinib or lenvatinib plus everolimus or sunitinib monotherapy as first-line treatment for mRCC (NCT02811861). Its preceding phase II trial revealed that pembrolizumab plus lenvatinib therapy offered an improved mPFS at 17.7 months (95% CI 9.6–N/A) as well as improved ORR of 66.7% (95% CI 47.2–82.7). The phase III three-armed expansion plans to recruit 735 treatment-naïve patients. The primary endpoint will be PFS with secondary endpoints as ORR, OS, health-related quality of life (HRQoL), and safety profiles. The CheckMate 9ER trial is a two-armed phase III randomized, open-label study exploring nivolumab plus cabozantinib versus sunitinib monotherapy. Interestingly, a recent phase I trial exploring this combination therapy demonstrated impressive anti-tumor activity but enrolled pretreated patients with mRCC [54]. CheckMate 9ER is now exploring the same combination in 630 previously untreated mRCC patients (NCT03141177) [106]. A last anti-PD-1 and VEGF TKI combinatorial regimen brings together pembrolizumab plus cabozantinib. Although these results are from the phase I setting, they are promising and this combination may have an impact on patient care in the future. Previously treated mRCC patients treated with combination therapy demonstrated encouraging early efficacy with an ORR 25% and a clinical benefit rate 87.5%. Enrollment of a phase II dose expansion is now ongoing (NCT03149822) [107].

There remain CPIs approved for other advanced tumors that have yet to establish a role in the treatment of mRCC. Tremelimumab is a CTLA-4 CPI that was examined in combination with sunitinib in a phase I dose-escalation trial with treatment-naïve patients. Of the patients evaluable for response, the ORR was 43% (95% CI 22–66%) and disease stabilization occurred in 33%. However, this study was halted due to unexpected and surprising TRAEs, including acute renal failure andeath [108]. The excitement for tremelimumab in mRCC has since diminished. Notwithstanding, tremelimumab remains under investigation in multiple settings with ongoing phase I trials; neoadjuvant tremelimumab in combination with durvalumab prior to nephrectomy (NCT02762006); and neoadjuvant tremelimumab monotherapy with and without cryoablation prior to nephrectomy (NCT02626130).

#### Novel CPI pathway inhibition

The field of immuno-oncology has unveiled a deeper understanding of the immunoreactivity inherent to mRCC, and investigators continue to identify new co-inhibitory ligands implicated in tumor immune evasion [109]. PD-1 and CTLA-4 receptor pathways belong to the B7/CD28 receptor family and have been the foundation for CPI drug discovery. However, our knowledge of the newer co-stimulatory and co-inhibitory pathways within this family remain rudimentary, and more insight into the receptor pathways within this family of receptors will no doubt offer other avenues for augmenting immune responses in the treatment of cancer [110]. Human endogenous retrovirus-H long terminal repeat-associating protein 2 (HHLA2) is a cell membrane and cytoplasmic protein involved in T cell activation and immune checkpoint blockade. Janakiram et al. has labeled HHLA2 as the third group of the B7-CD28 immune checkpoint family after PD-L1 and CTLA-4 [111, 112]. Chen and colleagues have recently demonstrated that HHLA2 has increased expression in ccRCC tumor tissue and that increased expression leads to a remarkably shorter OS and a poorer prognosis [113]. HHLA2 is emerging as a novel target for CPI therapies.

### Emerging novel tumor vaccines

Tumor vaccines (TVs) have been widely investigated and are being evaluated in an effort to make tumor cells more immunogenic and thereby overcome their immunosuppressing defense mechanisms [114, 115]. TVs are typically engineered with one of two approaches, via synthesis with dendritic cells (DCs) and tumor lysate, or via heat shock proteins [116]. The majority of vaccines in development and those with the most promise have employed DCs and RCC tumor lysate [78]. Functionally active DCs cells act as autologous tumor infiltrating lymphocytes which upregulate cytokine production within the tumor microenvironment with the aim of heightening the immune response within the tumor microenvironment [114]. IMA901 is such a vaccine, building off of nine different HLA-binding tumor-associated peptides which promote CD8^+^ and CD4^+^ T cell activation-mediated immune responses against malignant cells. The IMPRINT trial was a large phase III, multicenter, randomized control trial in which the IMA901 vaccine was combined with sunitinib in previously untreated mRCC patients [117]. Unfortunately, IMPRINT failed to demonstrate any difference in patient outcome compared to sunitinib monotherapy. A similar autologous DC-based vaccine, rocapuldencel-T, was successful with a beneficial effect in the phase II setting and was recently being tested in the phase III ADAPT trial. ADAPT has been suspended by Argos Therapeutics after findings of an interim analysis revealed the TV was unlikely to meet any of its primary endpoints [118].

As an alternative approach to standard TV development in RCC, immunologists have theorized that using allogeneic as opposed to autologous DCs will more likely potentiate a T helper 1-deviated inflammatory reaction, further promoting recruitment and activation of endogenous lymphocytes to the tumor [119]. INTUVAX is an allogeneic TV which has been successful in the phase I/II setting in 12 intermediate-and poor-risk patients with newly diagnosed mRCC. The trial was multifaceted and heterogenous in adjuvant treatments, but the results collectively suggest that intratumoral administration of proinflammatory allogeneic DCs induces an anti-tumor immune response that may prolong survival in unfavorable-risk mRCC [120]. INTUVAX is now being trialed in the randomized phase II MERECA study (NCT02432846).

## Prostate cancer

PC is the second most common cancer in men and the second leading cause of cancer death in the USA. A man’s risk of developing PC is 1 out of 9 [121]. Treatment of newly diagnosed PC depends on anatomic extent of disease, histologic grade, and serum prostate-specific antigen (PSA) level. Localized PC is often initially treated with either radical prostatectomy or radiation therapy. However, statistics show 27–53% of patients will develop biochemical recurrence [122]. Androgen receptors (AR) play a crucial role in the pathogenesis of PC and remain the key therapeutic target [123]. Androgen deprivation therapy (ADT), either surgical or chemical, has been the mainstay treatment for almost a century. Patients with a high PSA level, despite appropriate ADT, are diagnosed with castrate-resistant prostate cancer (CRPC) [124]. The average time of onset of castrate resistance after starting ADT is 19 months [125]. At this stage, the primary goal of treatment is to delay the time to metastasis. Current standard of care treatment of CRPC has been a well-established chemotherapeutic agent, docetaxel [126]. Although chemotherapy is effective in advanced PC, median survival remains less than 2 years. Due to the inevitable development of resistance, studies have remained rampant in exploring novel agents. As such, the standard of care has rapidly changed for PC within the past few years with the emergence of abiraterone, an androgen synthesis inhibitor, and enzalutamide, an androgen receptor antagonist. The STAMPEDE and LATITUDE trials were pivotal in assessing efficacy of abiraterone plus prednisone combined with ADT as first-line treatment in men diagnosed with metastatic castrate-sensitive prostate cancer (mCSPC). In both trials, significant improvement of PFS and OS was witnessed [127, 128]. The AFFIRM and PREVAIL trials led to the approval of enzalutamide for metastatic CRPC before or after docetaxel [129]. Thus, the standard of care has rapidly shifted for advanced PC over the past year. While these agents have had successful outcomes, resistance to treatment remains an inevitable reality for most patients. To this end, sequencing and combination of agents in PC has become a challenge. For mCRPC, first-line therapy has been established but there is a lack of data on which second- and third-line agents are most efficacious. Investigators have compared treatments in attempts to elucidate ideal sequences without clear data favoring any particular regimen [130]. Predictive biomarkers such as homologous repair mutations, mismatch repair mutations, and AR splice variants are beginning to emerge and will play a role in personalizing therapy. Ultimately, lengthy discussions with patients and consideration of various factors (i.e., disease volume, symptoms, age, functional status, cost) all help guide decision making in treatment design. The landscape of treatments for PC continues to evolve and a multitude of novel agents continue to emerge with ongoing trials showing great potential (Table 3).

**Table 3:** Emerging targets with clinical significance in PC

Molecular target	Class	Trial	Disease setting	Agent	Experimental treatment	Study phase	Estimated completion
Hormonal Therapy	Second-generation ADT	NCT03098836	mCRPC	Apalutamide	Apalutamide + abiraterone	Phase II	June 2021
		NCT02106507	mCRPC	Apalutamide	Apalutamide +everolimus	Phase I	April 2020
		NCT02489318 (TITAN)	mCSPC	Apalutamide	Apalutamide + ADT	Phase III	July 2022
	New-generation ADT	NCT02200614 (ARAMIS)	nmCRPC	Darolutamide	Darolutamide	Phase III	June 2020
	AR inhibitor	NCT02445976	CRPC Progressing on Enzalutamide or Abiraterone	Seviteronel	Seviteronel	Phase II	January 2019*
		NCT02012920	CRPC	Seviteronel	Seviteronel	Phase II	January 2019*
Tumor Vaccine	Dendritic Cells	NCT02111577 (VIABLE)	mCRPC	DCVAC	DCVAC	Phase III	June 2020
PD-L1 and CTLA-4	CPI	NCT02861573 (KEYNOTE-365)	mCRPC	Pembrolizumab + olaparib or Pembrolizumab + docetaxel + prednisone or Pembrolizumab + enzalutamide		Phase I	May 2022
		NCT02985957 (CheckMate-650)	mCRPC	Nivolumab + Ipilimumab		Phase II	March 2022
PD-L1		NCT02787005 (KEYNOTE-199)	mCRPC	Pembrolizumab	Pembrolizumab monotherapy or Pembrolizumab + enzalutamide	Phase II	December 2020
PARP Inhibitor	DNA Repair Inhibition	NCT03834519 (KEYLYNK-010)	mCRPC	Pembrolizumab + olaparib	Pembrolizumab + olaparib	Phase III	September 2022
		NCT03732820	Previously-untreated mCRPC	Abiraterone + olaparib	Abiraterone	Phase III	August 2022
Radioisotope		NCT03737370	mCRPC	Radium-223	Radium-223 + docetaxel	Phase I	October 2021
		ACTRN12615000912583** (LuPSMA Trial)	mCRPC	^177^Lutetium		Phase I/II	N/A

All trial information obtained through publicly accessible clinicaltrials.gov

*Results pending

**Trial filed in Australia New Zealand Clinical trials registry, http://www.anzctr.org.au

### Emerging hormonal therapy

The progressive nature of PC remains highly variable that can transform over many years. On average, castrate resistance develops 19 months after starting hormonal deprivation in non-metastatic PC [125]. Even in this scenario, multiple studies have shown a survival advantage with continuation of ADT [131]. Thus, the FDA approval of second-generation ADT agents, apalutamide (ARN-509) and enzalutamide (MDV3100), in non-metastatic CRPC in 2018 was a monumental feat for delaying metastatic disease [129]. Second-generation anti-androgens hold numerous advantages over first-generation agents: bicalutamide, milutamide, and flutamide. First and foremost, they hold a higher affinity for the AR, allowing greater efficacy in its antagonistic properties. Furthermore, second-generation anti-androgens do not have agonistic properties as observed in their first-generation counterparts, allowing fewer mechanisms of resistance [123, 126]. Enzalutamide is a well-established second-generation anti-androgen. Apalutamide, on the other hand, has recently risen to compete for standard of care therapy for CRPC. Apalutamide is a synthetic biaryl thiohydantoin compound that binds to the ligand-binding domain of the AR, with a seven- to tenfold increased affinity compared to bicalutamide [132, 133]. The SPARTAN trial, a double-blind, placebo-controlled phase III trial was pivotal for the approval of apalutamide. The primary endpoint measured metastasis-free survival (MFS), defined as the time from randomization to the first detection of distant metastasis on imaging or death from any cause [134]. The primary endpoint significantly favored the apalutamide group with an MFS of 40.5 months compared to 16.2 months in placebo, nearly a 2-year delay in metastasis [126]. Currently, the data is too premature to answer whether these drugs improve OS as only 24% of deaths occurred at time of publication. Shortly after the approval of apalutamide, the FDA also approved enzalutamide for non-metastatic CRPC [135]. Similar to the SPARTAN trial, enzalutamide also demonstrated extraordinary findings in the PROSPER trial. PROSPER had a primary endpoint of MFS which was 36.6 months in the enzalutamide group compared to 14.7 months in placebo in non-metastatic CRPC [136]. Due to the overwhelming evidence supporting second-generation anti-androgens, the landscape of advanced PC is rapidly evolving. There are a plethora of ongoing trials further testing second-generation anti-androgens in combination with many of the current mainstay treatments.

The addition of apalutamide to ADT in mCSPC has rendered promising results in the TITAN trial [137]. The trial’s co-primary endpoints, radiographic PFS and OS, were reportedly met; thus, the study was unblinded in January 2019 [138, 139]. Hence, apalutamide was submitted to the FDA for approval in April 2019 for mCSPC with the final study results presented at ASCO in 2019. The study met its primary endpoints, with a significant improvement in OS, with a 33% risk reduction in death [140]. Secondary endpoints also favored apalutamide with prolonged time before PSA progression and chemotherapy initiation. Interestingly, 10% of patients in the study had prior docetaxel exposure and those patients did not respond to apalutamide with ADT as well as the patients without docetaxel usage (95% CI 0.52–3.09). These results further strengthen the theory of different resistance mechanisms based on prior treatments, regardless of disease progressions. Furthermore, apalutamide is being studied in phase III trials as combination therapies; addition of apalutamide to abiraterone/prednisone and docetaxel, abiraterone, and everolimus are all underway (NCT03098836, NCT02106507) [141, 142]

Darolutamide (ODM-201) is another AR antagonist undergoing phase III clinical study to determine its efficacy in non-metastatic CRPC. Preclinical studies have shown increased anti-tumor activity compared to other second-generation anti-androgens, enzalutamide, and apalutamide. More specifically, darolutamide was studied in the vertebral cancer of the prostate xenograft model, which expresses high levels of AR wild type and of the V7 splice variant, and in the enzalutamide-resistant MR49F model which contains the AR mutations F877 L and T878A [143]. Results illustrated stronger antagonism when linked to AR mutants W742C and F877 L, which are resistant to enzalutamide and apalutamide. Of note, stronger antagonistic properties were also seen in the M896 T and M89 V forms, in which enzalutamide had reduced activity [144]. This potent AR antagonism is attributed to the chemical structure of darolutamide, AR ligand-binding via its isopropylamine linker and maintained van der Waals contacts with the leucine side of AR [145]. Furthermore, full antagonistic functionality of the AR is predicated on recruitment of its co-regulators. One of the co-regulator peptides include NCoR1, a corepressor that competes with AR antagonists attenuating agonistic activity [146]; PELP1, a member of chromatin remodeling complexes [147]; and TRXR1, which is upregulated in proliferating PC cells [148]. Darolutamide was shown to repel NCoR1 in the W742C mutant, which not evident when challenged with enzalutamide [145]. Once taken to clinical trials, darolutamide continued to show great potential. In the phase II ARAFOR trial, darolutamide demonstrated a 50% decrease in PSA levels from baseline in 83% of patients and was tolerated well [149]. Moving forward, the ARAMIS trial, an ongoing phase III double-blind, placebo-controlled trial, compared the safety and efficacy of darolutamide with placebo in non-metastatic CRPC patients. The primary endpoint in this study is MFS [150]. Final results were encouraging, indicating a MFS of 40.4 months in darolutamide group compared to 18.4 months in placebo group. The 3-year rate of OS was 83% in the darolutamide group versus 73% in the placebo group, conferring a 29% reduction in the risk of death (HR, 0.71; 95% Cl 0.50–0.99, *p* = 0.0452). mPFS was 36.8 months in the darolutamide group versus 14.8 months in the placebo group, attributing to a 62% risk reduction with darolutamide. The ARAMIS trial has raised darolutamide as a viable treatment choice for advanced PC.

Seviteronel (INO-464) is a selective CYP17 lyase (17,20-lyase) inhibitor, similar to abiraterone, but also has dual function as an AR inhibitor [151]. Seviteronel has a tenfold selectivity towards CYP17 lyase over hydroxylase and is a competitive antagonist in both the wild type and aforementioned mutated forms of the AR, T887A and F876 L [152]. The selectivity towards CYP17 lyase over hydroxylase renders seviteronel an advantage in avoiding effect on upstream steroids as seen with abiraterone. For example, although testosterone reduction is similar between seviteronel and abiraterone, abiraterone causes a significant increase in progesterone and corticosterone due to its increased 17-α-hydroxylase inhibition [153]. The potential resistance mechanism to abiraterone is progesterone-dependent stimulation of the AR with a T878A point mutation [154]. Thus theoretically, seviteronel’s lack of progesterone stimulation may aid in prolonging its affect and delaying resistance. In a phase I trial, men with CRPC, including those with prior exposure to abiraterone and/or enzalutamide, tolerated seviteronel well. 11 of 20 patients demonstrated a PSA decline (of any magnitude), four of whom had prior exposure to abiraterone and/or enzalutamide [155]. Seviteronel is currently being explored in several phase II studies of patients with CRPC who have developed resistance to current antihormonal therapies (NCT02130700, NCT02445976, and NCT02012920).

### Emerging immunotherapy

PCs exhibit evasive strategies to avoid detection and destruction by the immune system. While recent advances in immunotherapy have revolutionized the management of various solid and liquid malignancies, the impression it has left on the PC therapeutic landscape is nominal. Sipuleucel-T is the first FDA-approved immunotherapy for PC, and none have been approved since [156]. The two primary immune targeting approaches in ongoing PC research include antigen-targeted immunotherapy (i.e., vaccines) and CPI (CTLA, PD-1 inhibitors).

Sipuleucel-T is an autologous vaccine which triggers activation of antigen-presenting cells, mainly DCs, from signaling by a recombinant fusion protein, comprised of prostatic acid phosphatase (PAP) and granulocyte-macrophage colony-stimulating factor. These revamped DCs are then infused back into the patient and the vaccine generates CD4^+^ and CD8^+^ T cell responses against PAP, an antigen highly expressed in most PC cells [156]. In 2010, the IMPACT trial showed a 4.1-month improvement in OS compared to placebo in mCRPC [157]. Multiple trials are underway combining Sipuleucel-T with hormonal agents, chemotherapy, radiation, and other immunotherapy modalities. DCVAC/PCa is a promising vaccination strategy that is composed of activated DCs matched with killed LNCaP cells, a PSA-positive PC cell line. Both phase I and II trials revealed that a combination of DVCAV and cyclophosphamide given with docetaxel increased OS by 7.2 months compared to control [158]. A phase III trial is underway comparing the clinical efficacy of DCVAC to standard of care chemotherapy (NCT02111577). PROSTVAC-VF is a recombinant viral vaccine, which induces lysis of epithelial cells causing peripheral PSA release, which is absorbed by effector T cells. This cascade ultimately induces a PC-targeted immunogenic response. To further propagate the immunogenic response, the vaccine antigen is conjugated to co-stimulatory molecules B7.1, ICAM-1, and LFA-3 [159]. In a phase II trial, asymptomatic mCRPC patients had an improved OS of 25.1 months versus 16.6 months with vaccination therapy [160]. The PROSPECT study is an ongoing phase III trial investigating PROSTVAC with GM-CSF and its efficacy on survival (NCT01322490). Early studies combining vaccines with CTLA-4 inhibition have demonstrated potential efficacy [161]. Lastly, PC has shown to express low levels of PD-L1 and induction of PD-L1 has been theorized as a possible treatment approach. Thus, therapeutic vaccines which induce PD-L1 expression are under consideration [162].

Ipilimumab has been involved in two notable phase III trials, both of which were underwhelming and lacked significant improvement in OS. However, through genomic analysis of treated resected tumors, there was a finding of higher expression of PD-1, PD-L1, and VISTA on the treated PC tumor cells. Due to these findings, it is speculated that the tumor microenvironment continues to adapt after exposure to CPI inciting upregulation of immune checkpoints [163]. This theory is being tested in a phase II clinical trial that is underway assessing the efficacy of ipilimumab plus nivolumab in mCRPC (NCT02985957). Preliminary results released in early 2019 revealed that for patients with mCRPC who had disease progression despite second-generation hormonal therapy (cohort one), the ORR was 26% with combination immunotherapy at a median follow up of 11.9 months. Cohort two included those with progression of disease after chemotherapy and hormonal therapy, and these patients had an ORR of 10% at a median follow up of 13.5 months [164]. Although CPI monotherapy has had limited success in CRPC, ipilimumab plus nivolumab may have created a foundation for emerging immunotherapy therapies. Further studies are underway assessing the optimal sequence and timing of these CPIs.

Tremelimumab and pembrolizumab are other CPIs undergoing investigation. In a phase I trial, tremelimumab was combined with short-term ADT in patients with CRPC. Results were remarkable for prolongation of PSA doubling time, although no initial effect on PSA level [165]. In KEYNOTE-028, patients with PD-L1 expression in metastatic PC were treated with pembrolizumab monotherapy. All had prior treatment with docetaxel and targeted hormonal therapy. Results revealed an ORR of 13%, with a mDOR of 59 weeks and a stable disease rate of 39% [166]. To further evaluate the durability of CPI activity, metastatic PC patients were grouped based on PD-L1 expression and treated with pembrolizumab in the phase II KEYNOTE-199 trial (NCT02787005). Cohorts of patients who had RECIST-measurable PD-L1 positivity (C1) and negativity (C2) were grouped, as well as non-measurable, bone-predominant disease (C3). The latest preliminary results released in 2018 revealed ORR of 5% in C1 and 3% in C2. Median OS was 9.5 months in C1, 7.9 months in C2, and 14.1 months in C3 [167]. The trial is ongoing with more results to follow; however, initial results indicate a lack of difference between the groups, alluding that PD-L1 status alone may not be a sufficient targeted biomarker for a response.

Most recently, pembrolizumab has joined many other treatment combinations to further test its efficacy. KEYNOTE-365 is an open-label phase Ib/II umbrella trial evaluating four different treatment combinations, cohort A: pembrolizumab plus olaparib, cohort B: pembrolizumab plus docetaxel plus prednisone, cohort C: pembrolizumab plus enzalutamide (NCT02861573). Preliminary results were recently presented only for cohort A, pembrolizumab plus olaparib. Olaparib belongs to a family of poly-ADP ribose polymerase (PARP) inhibitors. PARP is a family of enzymes, activated by DNA damage, facilitating DNA repair via single-stranded break and base excision repair pathways. More specifically, PARP binds to single-strand DNA damage via its zinc-finger DNA-binding domain and recruits proteins involved in DNA repair via auto-poly(ADP-ribosyl)ation. This becomes a critical component for cancer cell survival [168]. PCs with DNA repair gene alterations have found to be sensitive to PARP inhibitors [169]. To this end, the PARP suppression in mCRPC first assessed in the TOPARP-A trial, which was significant for high response rates (88%) in patients with DNA repair gene deficits [170]. In an ongoing phase II trial, patients previously treated with docetaxel demonstrated a prolonged radiologic progression-free survival of 13.8 months with olaparib plus abiraterone versus only 8.2 months in the abiraterone monotherapy group [171]. Interestingly enough, even patients without homologous recombination repair also benefited from the combination therapy. Of note, there were more reports of TRAEs in the combination group compared to the control group. To build on the promising survival data, a phase III trial has commenced evaluating olaparib with abiraterone, but now as a first-line treatment for mCRPC (NCT03732820).

Yu and colleagues recently presented various treatment combinations with olaparib in 41 men in cohort A of the KEYNOTE-365 trial. These men were previously treated with second-generation hormonal therapy, chemotherapy, and docetaxel. Of the 28 patients with RECIST-measurable disease, 39% experienced a reduction in tumor burden. The ORR for the RECIST-measurable group was 7%. Overall, results showed a median OS of 13.5 months, PFS of 4.7 months, and PSA response of 12% [172]. Yu and colleagues are expanding the current study into a phase III trial, KEYLYNK-010, and will now be including patients who have also been previously treated with abiraterone and enzalutamide (NCT03834519).

### Emerging biomarker-guided therapy

Germline mutations in DNA damage repair (DDR) genes have garnered much attention among PC investigators. Studies have revealed a prevalence of various DDR defects in about 10% of primary tumors and almost 25% of metastatic tumors [173]. The majority of the germline or somatic aberrations in the DDR genes include BRCA1/2, CDK12, ATM, FANCD2, and RAD51C, with BRCA2 being the most common [174]. Further, the PROREPAIR-B study demonstrated that germline BRCA2 mutation carriers developed resistance to ADT quicker than non-carriers. The median time from initiation of ADT to CRPC was 28 months in non-carriers versus 13.2 months in carriers [175]. Not only is there evidence for quicker time to resistance, DDR mutation-positive (DDRm^+^) patients also suffer from shorted PFS rates [176].

At the 2019 ASCO conference, promising results of PARP inhibitors have in DDRm^+^ patients were presented. The previously mentioned TOPARP-A trial reported efficacy of olaparib in unselected metastatic mCRPC patients [170]. Thereafter, Mateo and colleagues conducted a phase II trial of olaparib in DDRm^+^ patients with mCRPC. The study consisted of 98 heavily pre-treated patients. Of note, the majority of patients had also been treated with abiraterone/enzalutamide. The overall radiological response was 54% in 400 mg-dose cohort, and 39% in 300 mg dose cohort. The mPFS was 5.4 months. Subgroup analysis revealed that BRCA1/2 had the highest response rate at 83% (mPFS 8.1 months), with the PALB2 defect group with the second highest response rate at 57% (mPFS 5.3 months), followed by ATM and CDK12 defects. Similarly, the BRCA1/2 and PALB2 groups also had the highest PSA 50%-fall response rates at 73% and 63%, respectively. Nearly 33% of olaparib-treated patients have not shown radiographic progression at the 1-year follow-up [177].

The development of new targeted strategies begs the question of the role of germline/somatic DDRm^+^ screening in all patients with advanced PC.

Next-generation sequencing studies reveal that 25% of metastatic PC patients harbor DDR defects, a prevalence significantly higher than previously recognized [178, 179]. Determination of DDRm^+^ patients is emerging as an essential step to successfully personalize treatment and could guide clinical decisions at key junctures in the course of PC treatment [180]. Widespread genetic testing remains cumbersome due to the high prevalence of PC in developed countries; however, future trial design incorporating DDR defect status will likely convey a survival advantage and further advance precision medicine outcomes.

### Emerging chemotherapy

For years, docetaxel with prednisone has been the well-established chemotherapeutic agents for CRPC [181]. Several studies have focused on other taxane agents but have failed to show significant improvement in OS. Thus, combination therapies involving docetaxel have become the mainstay of ongoing research in CRPC chemotherapy. Docetaxel is believed to have dual antineoplastic mechanisms: (1) inhibition of microtubular depolymerization and (2) attenuation of the effects of bcl-2 and bcl-xL gene expression. Enhanced microtubule stability leads to G(2)M phase arrest in the cell cycle and induces bcl-2 phosphorylation, eventually inducing apoptosis [182]. Studies had shown docetaxel to have a higher affinity for tubulin compared to other taxane agents such as paclitaxel. The combination of docetaxel to ADT was evaluated in the STAMPEDE trial, which was remarkable for improved PFS [183]. Thus, the addition of docetaxel for men who were committing to long-term ADT therapy became the standard of care. In the most recent studies, the combination of the two frontline agents for mCRPC, docetaxel and enzalutamide, has been under investigation (NCT02453009). Both have shown increased OS in their respective treatment regimens; however, little is known about the effects of the combination of the two. According to the preliminary results in the phase II CHEIRON trial, the combination of both had an improved 6 month PFS rate in patients with mCRPC compared to docetaxel alone. A 6-month PFS rate of 89% was observed in the combination group compared to 72.8% in the docetaxel monotherapy group. However, there has been no difference in median OS between the two groups [184]. Early docetaxel treatment concomitant with ADT has also been studied over the past years. The open label phase II CHAARTED study measured the efficacy of adding docetaxel plus ADT versus ADT alone. Results showed the mOS was 57.6 months in the combination study versus 47.2 months with ADT monotherapy [185]. However, the mOS in patients with high-volume disease was 51.2 months in the combination treatment group versus 34.4 months in the ADT monotherapy group. The authors concluded increased responsiveness in the subset of patients with high-volume mCSPC. Intriguing combination treatments with docetaxel are continuing to undergo investigation with multiple clinical trials underway.

Although platinum derivatives have failed to show OS benefit in patients with advanced PC, efficacy has been demonstrated in a distinct subset of patients [186]. Homologous recombination defects have been linked with increased sensitivity to platinum-based chemotherapy [187]. A recent case series revealed the effectiveness of carboplatin in three patients. Two of the three patients demonstrated BRCA2 and ATM mutations, respectively. Although BRCA2 mutation has been linked with poorer prognoses, the patient survived 15 years post-treatment compared to the reported 6-year median survival [188]. In an alternative report, eight men were identified with BRCA2 variants from a group of 141 men [180]. Six out of the eight men exhibited a PSA > 50% decline in 12 weeks compared to 23 of 133 non-carriers (95% CI 27–28%; *p* < 0.001). Although these reports have limitations, they allude to the potential of carboplatin in this subset of patients with advanced PC. These data are consistent with studies indicating increased responsiveness of BRCA2 carriers in the breast and ovarian cancer population to carboplatin.

### Emerging targeted therapy

Men living with mCRPC have a 90% rate of bone metastases, resulting in increased incidence of pathological fractures, spinal cord compression, and pain [189]. Activation of osteoblasts to increase bone mass and osteoclasts to resorb bone has been the primary mode of prevention. Zoledronic acid and denosumab successfully provided objective data on both HRQoL and prevention skeletal-related events; however, they have not been linked to increased OS [190]. Radium-223 is the first bone-targeting agent that has led to a significant survival benefit, both mPFS and OS [191]. Ongoing trials are evaluating the efficacy of radium-223 with other established agents linked with an increased OS; however, results to date have been underwhelming. Specifically, a randomized, double-blind, phase III trial compared concurrent use of radium-223 with abiraterone versus abiraterone (NCT02043678). Final results provided no skeletal event-free survival benefit with median symptomatic skeletal event-free survival of 22.3 months in the combination arm versus 26.0 months in the placebo group. More perplexing was that the incidence of fractures was 29% in the combination group and 11% in the placebo group [192]. Concurrent use of radium-223 and abiraterone is currently under further investigation but is not advised for clinical use at this time.

A promising bone-targeted therapy involves radiolabeled molecules bound to prostate-specific membrane antigen (PSMA), allowing targeted delivery of beta-radiation. PSMA is a 750 amino acid type II transmembrane glycoprotein [193]. It is thought to play a role in cell migration, nutrient uptake, and cell survival [194]. The levels of PSMA are low in normal prostate epithelium, however are found elevated 1000-fold in almost all PCs [194]. The PSMA receptor undergoes endocytosis when bound to its receptor proteins, allowing PSMA-labeled radioisotopes to concentrate within the cell [195]. ^177^Lutetium (^177^Lu) is a therapeutic radionuclide and a medium-energy β-emitter (490 keV) with a maximum energy of 0.5 MeV and a maximal tissue penetration of < 2 mm. The gamma rays emitted from ^177^Lu allows for visualization and localization of metastatic cancer cells [196]. In a phase II trial, 50 patients with mCRPC and PSMA positivity received four cycles of ^177^Lu-617 every 6 weeks (ACTRN12615000912583). An astonishing 64% observed PSA decline greater than 50%, and of those, 44% observed at least an 80% decline in PSA. Furthermore, of the 14 patients who did not undergo adequate PSA regression, 9 of them observed a PSA decline greater than 50% after a median of two subsequent cycles [197]. The correlation between PSA response and whole-body tumor dose was significant. Albeit a relatively small study, early results are promising and well tolerated in these patients who have already undergone standard of care therapy with docetaxel, abiraterone, and/or enzalutamide. More longitudinal investigation is needed to determine durability of response, but this is an exciting time for radiolabeled molecule targeting in PC.

## Discussion

We have now entered an era that has been characterized as “revolutionary” for therapeutic treatments in GU cancers. Over the past 20 years, we have come to recognize the necessity of understanding malignancy at the molecular level in order to better guide drug design and discovery [198]. We have made marked advances in our understanding of the molecular interplay within the tumor microenvironment. To this end, immunotherapy has emerged as possibly the most exciting oncological development of our generation, with CPI, TVs, and cytokine modulation providing objective and sometimes even sustained responses. Targeted therapy, predominantly via anti-angiogenesis, allow for a precision medicine approach that has also led to meaningful outcomes [198]. Moreover, the combination of immunotherapy and anti-angiogenesis has been validated as an approach that not only targets dual pathways of tumorigenesis but also functions in a synergistic approach enhancing each other’s therapeutic effects. As a result, we have witnessed a rapid evolution in the armamentarium of agents available for GU malignancies. There are ongoing paradigm shifts to therapy as treatment options are now being extended to the fourth- and fifth-line settings. There is no sign of stagnation in drug development as investigators are tooled with a deeper understanding of oncogenic drivers and targets. However, the rate by which novel therapeutic agents are being developed has not been reconciled by the literature with clear level one data to guide treatment choice. Study design has generally focused on proof-of-principle models and clinical efficacy of novel agents and not on head-to-head studies which would elucidate optimal sequence of agents. Nonetheless, the excitement and enthusiasm behind each emerging agent offers greater hope for clinical advances.

The treatment landscape for patients with advanced PC is continuing to evolve, now more than ever. STAMPEDE and LATITUDE were groundbreaking for abiraterone as the standard of care for mCRPC. The PREVAIL and PROSPER trials have been equally as groundbreaking for consideration of enzalutamide. Even with well-established drugs like enzalutamide and abiraterone, ongoing research for novel agents is essential. Although chemotherapeutic options are limited, novel hormonal agents such as apalutamide, darolutamide, and seviteronel are the upcoming frontrunners in battling castration resistance. The SPARTAN trial was pivotal and provided the framework for the FDA approval of apalutamide. The drug’s metastases-free survival rivaled that of the current frontline treatment of nmCRPC. Better understanding of abiraterone resistance has led us to the development of seviteronel. Seviteronel has shown superiority in certain AR mutant cohorts, potentially overcoming the barrier of resistance in some.

In the last year, immunotherapy has lost its status in advanced PC treatment. However emerging clinical trials are beginning to release promising preliminary results. Better knowledge of the tumor microenvironment, protein alterations, and treatment resistance mechanisms may further advance the role of immunotherapy in PC with better tailored drug design. The presence of PD-L1 expression is continuing to be explored in advanced PC. Trials combining immunotherapy with hormonal therapy and PARP inhibitors continue to grow and show great promise. Furthermore, identifying high-risk patients through gene sequencing can help delineate which subset of patients may most benefit from PARP inhibitors. Radiolabeled molecule targeting has been well tolerated and will likely have an important role in the future for CRPC.

With regards to UC and RCC, both can aptly be described as suffering a 20–30-year standstill of slow drug development with less than ideal clinical outcomes. Platinum-based chemotherapeutic agents continue their role in treatment cascades, albeit this role may be diminishing. CPI has had a transformative impact on patients with durable outcomes in a subset of individuals and with tolerable AE profiles. The approval of FGFR TKI erdafitinib provided new option for metastatic bladder cancer. Undoubtedly, combination therapies with dual immunotherapies, cytokine modulators, or with TKIs will be the focus on trial development for years to come. Combination therapy has the potential to overcome drug-resistance barriers as well as augment immunogenicity of the tumor—even in patients who lack significant response to CPI monotherapy. More exciting are the identification of novel molecular targets for CPI and the likely emergence of CPI therapies other than PD-1/PD-L1/CTLA-4 inhibitors. To this end, immunotherapy will have a strong foothold in the treatment landscape for both UC and RCC for years to come.

Both UC and RCC are at a crossroads for role of neoadjuvant cystectomy and cytoreductive nephrectomy (CN), respectively. CN in RCC was formerly the standard of care before the emergence of targeted therapies and CPI. Treatment paradigms transitioned over the past few years and the decision for CN is now more nuanced with consideration of MSKCC and IMDC prognosis scores [71]. The phase III CARMENA and SURTIME trials have provided concrete data on the effects of CN in various RCC populations. CN is only recommended in patients with a good-to-intermediate prognosis. However, this dogma is challenged by many experts and argues that CN should be considered on an individualized basis and that overarching trials offer RCC patients a disservice by excluding patients who may benefit from CN. There remains room for better trial design and further investigation of the clinical benefits (or lack thereof) for CN. Bladder preservation therapy (BPT) is an emerging concept that is gaining traction in UC. Neoadjuvant chemotherapy followed by radical cystectomy is the standard of care for MIBC. BPT has had favorable outcomes in ongoing studies. However, with the approval of CPI in the first-line setting in UC, large-scale clinical trial investigation of BPT with CPI remains an urgent unmet need. Early phase I/II single-arm trials are underway and will likely provide the biologic rationale for more expansive studies soon.

The pace by which we have identified novel therapeutic targets and the emergence of novel agents in GU oncology has been unprecedented. However, we are just entering the era of personalized medicine. We now appreciate that race, gender, and age all affect tolerability of various agents between individuals [199–201]. Early studies have demonstrated the power of genetic profiling as an invaluable tool with implications in diagnoses, therapeutics, and prognosis of cancers [78]. Further, leaders in the field have suggested that using next generation tumor sequencing to assess for genomic alterations may aid in treatment selection and should be considered [16]. Ghatalia and colleagues presented an elegantly designed study in which 35 patients with RCC underwent gene expression profiling. The study paired intrapatient kinase gene-expression analysis in primary dormant RCC, matched normal kidney, and mRCC and identified novel drivers of metastasis [202]. Alternatively, several studies have conducted gene analyses and found a predictive role for certain gene signature profiles. A 25-gene IFN-γ gene expression signature has been correlated with nivolumab responsiveness in UC [19]. A commercially produced NanoString gene expression platform specific to an 18-gene signature has been shown to effectively predict pembrolizumab responsiveness [203]. Altogether, gene expression profiling provides a platform for high-throughput genetic evaluation of patient tumors and is an exemplary example of the impact personalized may have in the future of GU oncology. Pre-determination of responsiveness to CPI would be invaluable for patient selection for immunotherapy. Lastly, studies experimenting with GU circulating tumor cells, circulating tumor DNA, and tumor organoids have been a novel approach to harness genomic data from in vivo cancer specimen to create a “personalized” genomic panel [204–206]. Prospective clinical trials are warranted to further validate these technologies as a tool for personalized therapy.

## Conclusion

In conclusion, major advances in our understanding of GU cancer biology have had a transformative impact in the field. The emergence of novel agents, particularly immunotherapeutics, are having a profound impact in the field. The future is bright for GU oncology and continued validation of biomarkers in conjunction with combination therapies will likely optimize the efficacy of our treatments. We have highlighted the data behind emerging agents, and we will continue to learn the strengths and weakness as the novel agent’s trial results mature. Validation of molecular signatures and biomarker expression will be essential to stratify respective patients for proper treatment courses. Ultimately, investigators will be challenged with the task of sequencing ideal treatment cascades and to provide consensus agreements on optimal drug selection for each disease setting. This has proven a challenge in the current treatment landscape and will likely become more challenging with the rapid rate of drug development. Nonetheless, this is a welcomed challenge as PFS and OS continue to improve in this exciting time.

## Data Availability

Data sharing not applicable to this article as no datasets were generated or analyzed during the current study.

## Abbreviations

^177^Lu: ^177^Lutetium
ADC: Antibody-drug-conjugate
ADT: Androgen deprivation therapy
AE: Adverse event
ALK: Activin receptor-like kinase
AR: Androgen receptor
ASCO: American Society of Clinical Oncology
ccRCC: Clear cell renal cell carcinoma
CN: Cytoreductive nephrectomy
CPI: Check point inhibitor
CR: Complete response
CRPC: Castrate-resistant prostate cancer
DC: Dendritic cell
DDR: DNA damage repair
DDRm^+^: DNA damage repair mutation positive
FDA: Food and Drug Administration
FGF: Fibroblast growth factor
GU: Genitourinary
HDIL-2: High-dose interleukin-2
HER: Human epidermal growth factor receptor
HHLA2: Human endogenous retrovirus-H long terminal repeat-associating protein 2
HR: Hazard ratio
HRQoL: Health-related quality of life
IDO: Indoleamine-2,3-dioxygenase
IFN-α: Interferon-alpha
IL-2: Interleukin-2
mAB: Monoclonal antibody
mCSPC: Metastatic castrate-sensitive prostate cancer
mDOR: Median duration of response
MFS: Metastasis-free survival
MIBC: Muscle-invasive bladder cancer
mRCC: Metastatic renal cell carcinoma
mTOR: Mammalian target of rapamycin
nccRCC: non-clear cell renal cell carcinoma
NK: Natural killer
ORR: Objective response rate
OS: Overall survival
PAP: Prostatic acid phosphatase
PARP: Poly-ADP ribose polymerase
PC: Prostate adenocarcinoma
PFS: Progression-free survival
PSA: Prostate serum antigen
PSMA: Prostate-specific membrane antigen
RCC: Renal cell carcinoma
TK: Tyrosine kinase
TKI: Tyrosine kinase inhibitor
TNF: Tumor necrosis factor
TRAE: Treatment-related adverse event
Treg: T regulatory
TV: Tumor vaccine
UC: Urothelial carcinoma
VEGF: Vascular endothelial growth factor
